# Kinetics of skin temperature in lower limbs of professional soccer athletes

**DOI:** 10.5114/biolsport.2025.145909

**Published:** 2025-01-13

**Authors:** Alex de Andrade Fernandes, Miller Gomes de Assis, João Carlos Bouzas Marins, André Gustavo Pereira de Andrade, Maicon Rodrigues Albuquerque, Ciro José Brito, Cristiano Diniz da Silva, Myrian Augusta Araujo Neves do Valle, Eduardo Mendonça Pimenta, Emerson Silami Garcia

**Affiliations:** 1 Federal Institute for Education, Sciences and Technology of Minas Gerais (IFMG), Campus Ipatinga, Brazil; 2 School of Physical Education, Physiotherapy and Occupational Therapy – Federal University of Minas Gerais, Brazil; 3 Department of Physical Education, Human Performance Laboratory, Federal University of Viçosa, Brazil; 4 Department of Physical Education, Federal University of Juiz de Fora, Campus Governador Valadares, Brazil

**Keywords:** Soccer, Infrared thermography, Skin temperature, Creatine kinase, Inflammation

## Abstract

This study investigated the kinetics of skin temperature (Tsk) in the lower limbs of elite soccer players following official matches, with measurements taken at three time points: pre-game (M1), 24 hours after a match (M2), and 48 hours after a match (M3). Additionally, we explored the correlation between Tsk and individualized creatine kinase (CK) levels. Thirty male athletes from a top-tier professional soccer club were assessed during the Brazilian Serie A Championship. CK levels and Tsk in the lower limbs were recorded at M1, M2, and M3. Tsk was significantly elevated at M2 compared to M1 (P < 0.001) and decreased at M3, although it did not return to baseline levels (P < 0.001). A significant positive correlation was found between Tsk in all regions of interest (ROIs) and the percentage of CK, with the lowest correlation observed at r = 0.52 (P < 0.001). Tsk in the lower limbs showed a pattern similar to CK, being elevated at 24 h after the match and decreasing by 48 h but not fully returning to pre-game levels. These findings suggest that Tsk can complement CK measurements and be useful in training control and recovery strategies for elite soccer athletes.

## INTRODUCTION

Infrared thermography (IRT) captures infrared radiation emitted by individuals and translates it into visible thermal images, providing temperature readings. Recent years have seen a growing interest in utilizing infrared thermography (IRT) to monitor skin temperature (Tsk), and the results suggest that this approach holds significant potential as an effective athlete monitoring strategy [1–3].

Soccer involves intense motor actions with eccentric contractions [2, 4], leading to muscle damage and local inflammatory responses characterized by phagocyte infiltration, elevated interleukin-6 (IL-6) and creatine kinase (CK), and synthesis of acute-phase proteins such as C-reactive protein (CRP) [2, 4]. CK serves as an indirect marker of exercise-induced muscle damage [5, 6], providing information on post-exercise recovery status. However, routine CK monitoring in soccer clubs may benefit from supplementary strategies [7, 8].

The serum concentration of CK is acknowledged as an indirect marker of exercise-induced muscle damage [5, 6]. Therefore, assessing CK levels can provide valuable information about an athlete’s recovery status after strenuous physical activity. Serum CK concentrations can be influenced by various variables, such as age, gender, genetic predisposition, and training period [5, 9]. Moreover, the method of result analysis is also crucial, as different soccer players exhibit various thresholds for CK levels. Therefore, an individualized CK concentration profile should be used to monitor each soccer player throughout a championship [10].

Tsk can be a variable associated with muscle damage caused by matches and training sessions, allowing it to be used as a training control strategy in high-performance soccer athletes [1, 11–14]. Nonetheless, it is still unclear whether Tsk, measured by IRT, could be an outcome related to the effects of cumulative fatigue [15–17]. Furthermore, variations in analysis conditions and the studied population may lead to divergent results. Thus, further studies are essential to contribute to the scientific progress of sports sciences.

Thus, the aim of this study was to analyse the kinetics of Tsk in the lower limbs of elite soccer athletes after participating in official matches at pre-game, 24-hour, and 48-hour post-game time points. The study also sought to investigate the potential correlation between Tsk and the individualized percentage of CK and compare the regions of interest (ROIs) on the right side with the left side, as well as the anterior view with the posterior view.

## MATERIALS AND METHODS

### Ethical considerations

The experimental procedures of this study were approved by the Research Ethics Committee of the Federal University of Minas Gerais, with a registration number in the Certificate of Presentation for Ethical Consideration (CAAE): 50708915.6.0000.5149. This study complied with the regulations established by Resolution 196/96 of the National Health Council, as well as the principles outlined in the Declaration of Helsinki by the World Medical Association. All procedures, potential risks, and benefits of the study were explained to the participants, who then provided informed consent by signing a consent form to participate in the study.

### Participants

The convenience-based, casuistic sample consisted of 30 professional soccer athletes from a first-division club, who participated in systematic and regular training sessions and competed in organized and/or recognized competitions by the Brazilian Soccer Confederation (CBF), such as the Brazilian Soccer Championship – Serie A, Brazilian Cup, as well as continental competitions such as the CONMEBOL Libertadores and CONMEBOL Sudamericana.

For the present study, the following inclusion criteria were adopted: a) aged between 20 and 34 years; b) ≥ five (5) years in systematic soccer training; c) training for competition. We excluded those: a) who were already injured before the start of the study; b) who did not play the full game; c) suffered contact injuries in the lower limbs during the game; and d) for whom there were failures in the capture of thermographic images or in the analysis of CK. Our casuistic sample was composed of athletes who trained about six days per week, with approximately nine training sessions lasting around 2 hours each, in addition to the competition matches.

The sample size was determined using GRANMO software version 7.12 (available at https://www.datarus.eu/aplicaciones/granmo/), based on a previous study measuring creatine kinase in soccer players [6, 10], which was the primary variable of interest in this study. We set the significance level at 0.05 (two-tailed) with a 95% confidence interval, aiming for a power of 0.5 and an effect size of 0.2 to detect a difference of 50 IU/L between moments M1, M2, and M3. The calculations suggested a need for 26 participants, plus an additional 4 to account for a 10% loss rate. Prior to the study, all participants underwent a health evaluation and were confirmed to be healthy and physically fit for exercise.

### Characterization and physical assessment of participants

At this stage, a qualified physical education professional conducted an anthropometric assessment, which included measurements of body mass, height, and skinfold thickness. The study group’s mean age, body mass, height, skin fold, and maximal oxygen consumption are presented in Table 1. Body mass was measured using a digital scale (Filizola) with a precision of 0.02 kg. Height was measured using a stadiometer with a precision of 0.5 cm attached to a scale (Filizola).

**TABLE 1:** Characterization and physical assessment of participants.

	Mean	SD	Minimum	Maximum
Age (years)	27.2	± 4.4	20	34
Height (cm)	178.3	± 6.3	168	185
Body mass (Kg)	69.8	± 6.6	60	78
Skin fold (%)	9.8	± 3.0	6.4	14.1
$\dot{\text{V}}{\text{O}}_{2\max}$ (mL · kg^-1^ · min^-1^)	54.2	± 2.5	50.3	60.5

The group’s maximal oxygen consumption ($\dot{\text{V}}{\text{O}}_{2\max}$) was indirectly assessed using the YoYo Endurance Test (level 2) conducted during the preseason. The test was conducted in the late afternoon on natural turf, with all athletes wearing soccer cleats.

### Characterization of the matches

To analyse the intensity of the matches and the players’ movements, a GPS device (GPSports Systems, Canberra, Australia) with a sampling frequency of 15 Hz and a triaxial accelerometer of 100 Hz was used. Each player wore a GPS device (mass: 76 g; dimensions: 48 mm × 20 mm × 87 mm) placed on the back of a vest near the thoracic region.

### Experimental procedures

Data collection was conducted throughout the 2015 season in seven matches and the 2016 season in six matches of the Brazilian Soccer Championship – Serie A. All data collection was performed when the club was playing at home, and there was no need to travel for away games in subsequent matches.

The Tsk and CK data were collected at three different time points: M1 – Pre-game, 24 hours before the match; M2 – 24 h, 24 hours after the match; M3 – 48 h, 48 hours after the match, always in the afternoon and compatible with the match time, as Tsk can vary throughout the day. Only athletes who participated in at least 75% of the match’s playing time were evaluated and considered. During the matches, the participants were allowed to drink water ad libitum or isotonic beverages according to the coaching staff’s instructions.

For Tsk measurement, a thermal camera (Flir, T420, Stockholm) was used, with a measurement range of -20 to +120°C, accuracy of 1%, sensitivity ≤ 0.05°C, infrared spectral range of 7.5 μm to 13 μm, a refresh rate of 60 Hz, autofocus, and resolution of 320 × 240 pixels.

The procedures for Tsk data collection were the same for all participants at all time points, and were previously established in different studies [18, 19]. The data were collected in a room with properly controlled environmental conditions, where the average room temperature was 24.5 ± 0.3°C and the corresponding relative humidity was 51.4 ± 2.7%, indicating a temperate environment.

The acclimatization time for each athlete in the assessment room was 15 min. A questionnaire on the preceding conditions that could interfere with Tsk was administered before each analysis. Participants were instructed to avoid alcoholic beverages, smoking, caffeine, large meals, ointments, cosmetics, and showering for 4 h before the assessment. In addition, sunbathing (e.g., UV sessions or direct sun without protection) was avoided before the assessment. Extrinsic factors affecting skin temperature (e.g., massage, electrotherapy, ultrasound, heat or cold exposure, cryotherapy) were also avoided.

Starting from the anatomical position, the participant faced the thermal camera at a distance of 3 m for infrared thermography measurements. One thermal image was collected from the anterior view of the lower limbs and one from the posterior view. The club’s coaching staff, who were properly trained and experienced in this type of procedure, always performed these measurements, and the researchers supervised them at different times during the study.

**FIG. 1:**
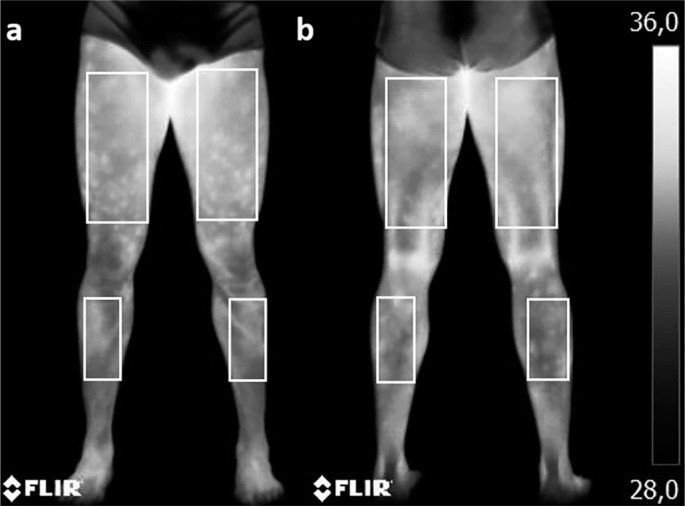
ROIs delimited, in this case, in an athlete at the M2.

ROIs rectangles were selected using specific software (Flir Tools), with dimensions of 10 cm width × 20 cm height for the thighs and 7 cm width × 19 cm height for the legs (Figure 1), as described before [1, 13, 20]. The emissivity value adopted for human skin was 0.98, and the reflected temperature from the room was 23°C.

To determine the enzymatic concentration of plasma CK, the digital pulp of the participants was cleaned with 95% ethanol, and then 32 μL of capillary blood was collected. After drying with cotton, an auto-trigger lancet was used for puncture, and the blood was drained into a heparinized capillary tube (Reflotron) and immediately pipetted onto a CK reagent strip (Reflotron) and inserted into the Reflotron Analyser. This collection was always performed by members of the club’s technical staff who were trained and experienced in this type of procedure, accompanied by researchers at various stages of the study.

The individual analysis of %CK followed the proposal of Alves [10], according to which it was necessary to know that the values of resting CK (CK_rest_) were obtained during the soccer players’ presentation after a 30-day rest from any physical activity. Game CK (CK_Game_) value were collected after matches during the competitive period, with the highest value found referred to as maximum CK (CK_max_).

### Statistical analysis

All variables were tested for normality using the Shapiro-Wilk test and were normally distributed. Results are presented as mean ± SD. One-way ANOVA for repeated measures, followed by Tukey’s post-hoc test, was used to compare Tsk values among all ROIs in the right and left legs, at moments 1 and 3, as well as for CK values at the three moments. Pearson’s correlation coefficient was used for Tsk and CK variables. The paired t-test was used to compare ROIs between the right and left sides and anterior and posterior views. The significance level was set at α< 0.05. All analyses were performed using the statistical software programs SigmaPlot 12.0, SPSS 17, and MedCalc 13.

## RESULTS

Table 2 provides a description of the information characterizing the matches analysed in this study, such as time, ambient temperature, relative humidity, total distance covered, metres per minute, and the number of actions above 25 km/h.

**TABLE 2:** Characterization of the matches analyzed in the study.

Game	Time	Temperature	Humidity	Distance (m)	Meters/min	Actions > 25 km/h
Game 1	16:00	28° C	75%	10125 ± 986.2	103.3 ± 10.0	7 ± 4.0
Game 2	16:00	27° C	72%	10234 ± 991.5	110.2 ± 9.1	8 ± 3.6
Game 3	19:00	25° C	79%	10951 ± 834.1	99.4 ± 11.2	7 ± 4.2
Game 4	17:00	31° C	70%	9457 ± 1053.3	97.7 ± 10.9	7 ± 3.4
Game 5	18:30	26° C	69%	1268 ± 916.3	93.5 ± 9.6	6 ± 3.2
Game 6	16:00	26° C	68%	9921 ± 1211.6	109.3 ± 10.1	7 ± 4.1
Game 7	16:00	28° C	74%	10324 ± 979.1	102.5 ± 10.6	6 ± 4.9
Game 8	19:00	27° C	75%	9874 ± 1010.6	102.5 ± 10.6	6 ± 4.9
Game 9	18:30	25° C	72%	10132 ± 1102.9	111.7 ± 9.9	6 ± 4.9
Game 10	16:00	22° C	73%	9698 ± 1112.8	104.6 ± 10.9	7 ± 3.5
Game 11	16:00	24° C	75%	10944 ± 988.8	108.5 ± 10.1	8 ± 3.9
Game 12	16:00	22° C	76%	10698 ± 1051.2	100.3 ± 10.4	7 ± 3.3
Game 13	19:00	27° C	74%	10509 ± 864.7	109.1 ± 9.5	7 ± 5.0

The matches analysed in this study were characterized by various parameters. The average ambient temperature during the games averaged 26.46 ± 2.28°C. The relative humidity during the matches was approximately 72.85 ± 2.96%. In terms of physical performance, the players covered an average total distance of 10068 ± 894.2 m, with an average speed of 103.02 ± 7.7 m/min. Additionally, there were an average of 6.62 ± 0.7 actions per match where the players exceeded the speed of 25 km/h.

**FIG. 2:**
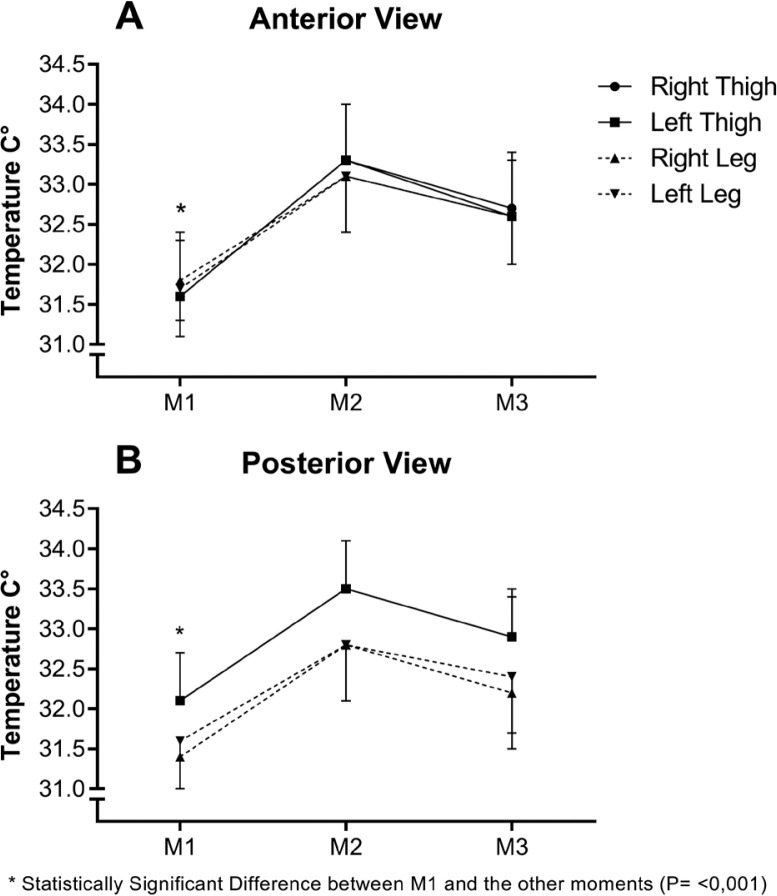
Mean Tsk in the RCIs throughout the different moments of the study, in the anterior view (A) and posterior view (B). M1 – Pre-game, 24 hours before the match; M2 – 24 h, 24 hours after the match; M3 – 48 h, 48 hours after the match

Figure 2 shows the mean Tsk values observed in different ROIs at different time points in the study. Table 3 presents the Tsk values of the thighs and legs (right and left) in the anterior and posterior views in°C, at different time points, as well as the delta (Δ) Tsk in the comparison of ROIs laterality and the delta (Δ) Tsk in the comparison between anterior and posterior views. Table 4 presents the correlation values of Tsk recorded in each analysed ROI with the individualized percentage of CK, following the proposal of Alves [10], at different time points.

**TABLE 3:** Mean Tsk of the thighs and legs (right and left) in the anterior and posterior views in°C, at different analyzed time points, as well as the delta (Δ) Tsk in the comparison of ROIs laterality and the delta (Δ) Tsk in the comparison between anterior and posterior views.

	Thigh R.	Thigh L.		Leg R.	Leg L
**AV**	**Tsk**	**Tsk**	**ΔTsk**	**Tsk**	**Tsk**	**ΔTsk**
**M1 – Pre**	31.6 ± 0.8	31.6 ± 0.7	0.0	31.8 ± 0.5	31.7 ± 0.6	0.1
**M2 – 24 h**	33.3 ± 0.7	33.3 ± 0.7	0.0	33.1 ± 0.7	33.1 ± 0.7	0.0
**M3 – 48 h**	32.6 ± 0.7	32.6 ± 0.7	0.1	32.6 ± 0.6	32.6 ± 0.6	0.0
**PV**						
**M1 – Pre**	32.1 ± 0.6	32.1 ± 0.6	0.0	31.4 ± 0.7	31.6 ± 0.6	0.2
**M2 – 24 h**	33.5 ± 0.6	33.5 ± 0.6	0.0	32.8 ± 0.7	32.8 ± 0.7	0.1
**M3 – 48 h**	32.9 ± 0.5	32.9 ± 0.6	0.0	32.2 ± 0.7	32.4 ± 0.7	0.2
**ΔTsk M1 AV-PV**	-0.5 ± 0.2*	-0.5 ± 0.2*		0.4 ± 0.2*	0.1 ± 0.1*	
**ΔTsk M2 AV-PV**	-0.2 ± 0.1*	-0.2 ± 0.1*		0.3 ± 0.1*	0.3 ± 0.1*	
**ΔTsk M3 AV-PV**	-0.3 ± 0.2*	-0.3 ± 0.1*		0.4 ± 0.1*	0.2 ± 0.1*	

**AV:** Anterior View; **VP:** Posterior View; **M1** – Pre-game, 24 hours before the match; **M2** – 24 h, 24 hours after the match; **M3** – 48 h, 48 hours after the match;

* *Sig. Est. Diff. (P < 0.001)*

**TABLE 4:** Linear correlation of Tsk recorded in each analyzed ROI with the individualized percentage of CK.

Thighs Right	Thighs Left	Leg Right	Leg Left
**Anterior View**	0.53*	0.55*	0.54*	0.52*
**Posterior View**	0.58*	0.57*	0.55*	0.53*

* Statistically significant correlation, P = 0.01

In the anterior view, the delta (Δ) of Tsk between M1 and M2 in the right and left thighs showed an average increase of 1.7°C (± 0.7°C), followed by increases of 1.1°C (± 0.6°C) and 1.0°C (± 0.6°C), respectively, between M1 and M3. In the right and left legs, the increase was 1.3°C (± 0.6°C) and 1.4°C (± 0.6°C) between M1 and M2, and 0.8°C (± 0.5°C) and 0.9°C (± 0.5°C) between M1 and M3, respectively.

In the posterior view, the delta (Δ) of Tsk between M1 and M2 in the right and left thighs showed an average increase of 1.5°C (± 0.7°C) between M1 and M2, followed by 0.9°C (± 0.6°C) in both thighs between M1 and M3. The right and left legs exhibited a difference of 1.4°C (± 0.7°C) and 1.3°C (± 0.7°C) between M1 and M2, respectively, and 0.9°C (± 0.6°C) and 0.8°C (± 0.6°C) between M1 and M3.

**FIG. 3:**
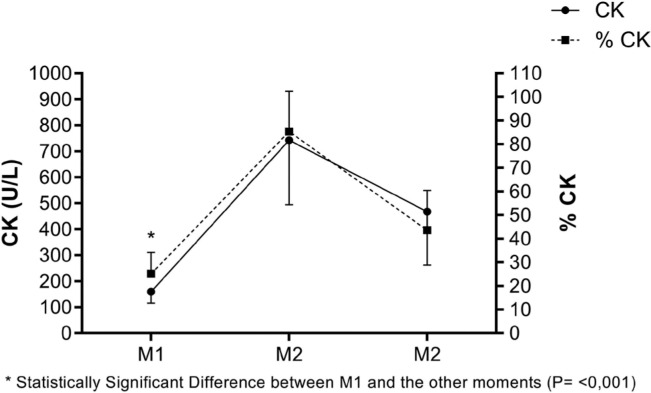
CK values (U/L) and their individualized % at different time points. M1 – Pre-game, 24 hours before the match; M2 – 24 h, 24 hours after the match; M3 – 48 h, 48 hours after the match.

Meanwhile, Figure 3 displays the values of CK (U/L) and the individualized percentage of CK, following the proposal of Alves [10], at different moments.

## DISCUSSION

This study provides insights into lower Tsk kinetics in professional soccer players following official matches over two seasons. Significantly elevated Tsk values across all analysed ROIs were observed at 24 hours after the match, followed by a notable decrease at 48 hours, albeit not returning to pre-match levels (Table 3 and Figure 2). These findings enhance our understanding of professional soccer players’ post-match physiological responses, underscoring Tsk as a valuable metric for monitoring training load.

In this study, a significant moderate positive correlation [21] was found between Tsk measured in each analysed ROI and the individualized percentage of CK (Table 4). This supports Tsk as a valuable metric for monitoring training load in soccer athletes. Despite its limitations, CK remains widely used for indirectly assessing post-match muscle damage.

Our findings contrast with Bandeira el al. [22] and Korman et al. [17], who reported no correlation between the variables. The differences may stem from the Bandeira et al. [22] methodology, which included only two data collection points (rest and 24 hours post-exercise) and intensities of exercise (low vs. high); furthermore, this protocol used raw CK values in U/L. We believe that further research is necessary to explore the potential relationship between CK and Tsk, particularly through protocols that involve long-term monitoring. Our findings align with Maior’s study [23], which, using an acute protocol, identified a weak correlation between Tsk and CK, likely due to data being collected during rest and reliance on raw CK values in U/L. In contrast, Korman et al. [17] observed an increase in CK over a 10-day period and a simultaneous decrease in Tsk, but found no correlation between these variables. Additionally, a metaanalysis of 13 studies by Dos Santos et al. [24] concluded that there was no significant correlation between CK and Tsk.

Priego-Quesada et al. [25] found that baseline skin temperature was not elevated at 24 and 48 hours after a marathon. They suggested that this lack of change could be due to muscle damage or inflammation not being near the skin’s surface. Conversely, conditions leading to peripheral vasoconstriction could enhance muscle vasodilation. This explanation suggests a curvilinear inverted U relationship between internal load and skin temperature responses.

Côrte et al. [12], who aimed to assess thermography’s role in preventing muscle injury in professional soccer players, took images twice weekly (48 hours after a game). The authors expected Tsk values to return to baseline at this point but observed asymmetries between ROIs ranging from 0.5°C to 1.5°C, prompting intervention. The images showed that 48 hours after the game, athletes’ Tsk levels were higher than baseline expectations.

When analysing the correlation between Tsk and CK, it is important to bear in mind that CK levels are highly variable and influenced by muscle damage across any body region. Therefore, if an athlete sustains an injury outside of the exercised muscles, it may result in higher CK values unrelated to muscle wear from exercise. Environmental conditions during matches, which are uncontrollable, can also affect CK measurements in official settings.

It is worth noting that this study removed outlier data points, which may reduce correlation values between Tsk and CK. Additionally, CK provides a global measure of muscle damage, whereas Tsk is valuable for pinpointing localized areas of hyperthermia.

## Pre-match analysis – M1

Tsk values across all analysed ROIs are presented in Table 3 and Figure 2. Comparing these findings with Bouzas Marins et al. (2014), who studied sub-19 soccer players from a first division Brazilian club, professional athletes showed higher Tsk levels, approximately 1.5°C higher in thighs and legs in the anterior view, and around 2.0°C higher in the posterior thigh. These differences may be attributed to factors such as higher training intensity, match frequency, as well as differences in muscle maturation and morphology, including smaller muscle cross-sectional areas in younger athletes. Additionally, thermal equilibrium was observed between contralateral ROIs, indicating consistent thermal patterns across the body segments analysed.

Compared with Maior [23], who studied professional soccer players from a first division Brazilian club, our results show relatively similar findings, with a slight decrease of approximately 0.3°C in all ROIs, except for the anterior thighs. These similarities can be attributed to both studies focusing on first division professional athletes. However, minor differences are expected due to variations in data collection timing, training phases, and inter-subject variability.

Regarding CK analysis, the obtained value at this time point, 159.5 U/L (Figure 3), is slightly lower than the mean value of 192.1 U/L reported by Coelho [26], and comparable to those reported by Mohr [27]. This suggests consistency in CK levels among trained athletes in competitive conditions.

## 24 hours post-match analysis – M2

This study showed a significant increase in Tsk across all analysed ROIs of the lower limbs 24 hours after matches, with peak values observed at this time (Figures 1 and 2, Table 3). Differences in Tsk compared to pre-match values (M1) exceeded 1.3°C across all ROIs, reaching 1.7°C in the anterior thigh regions. Notably, some athletes exhibited differences exceeding 3.0°C without clinical signs of fever or injury, as per medical evaluation. Compared to normal individuals and young athletes at rest [20, 28], these differences exceed 3.0°C at this time point.

Compared to professional athletes, our study observed temperature differences of approximately 1.5°C in the anterior thighs and legs, and about 2.0°C in the posterior thigh, as noted by Maior et al. [23]. These findings suggest a potential influence of inflammatory processes during muscle tissue regeneration on increases in Tsk. However, caution is advised in interpreting these results, as future studies may either corroborate or refute our findings.

Regarding CK analysis, our research recorded values of 743 U/L (Figure 3), slightly lower than Coelho [26], with 785.8 U/L on average, yet higher than Mohr et al. [27]. Tsk shows a similar trend to CK analysis, whether in raw U/L values or individualized percentages (Figure 3). Unlike CK analysis, which offers a broad assessment of muscle damage, Tsk can pinpoint specific muscle locations with acute micro-injuries and inflammation after training or a match [12]. This capability allows early detection of minor muscle imbalances, crucial for injury prevention programmes. In soccer, this proactive approach can lead to significant economic benefits by preventing long-term injuries that might make players miss matches.

## 48 hours post-match analysis – M3

At this juncture, Tsk readings began to approach pre-match levels. However, 48 hours after a match proved insufficient to fully restore the physiological conditions noted at M1 (Figures 1 and 2). The ongoing inflammatory process in the active musculature likely accounts for this observation, resulting in elevated Tsk values. Nevertheless, a declining trend in inflammatory activity by this stage contributed to subsequent lower Tsk values.

This decline suggests to coaching staff that the athlete is beginning to recover. Consequently, activities can be scheduled to avoid introducing new high-intensity physical loads, ensuring complete recovery. If Tsk values remain similar to those observed at the 24-hour mark, extending the recovery period is advisable to ensure full physical recuperation and potentially prevent injuries.

Similarly to the findings of Fernandes [1], this study observed differences ranging from 0.2°C to 0.7°C compared to pre-match values, varying by ROI. Differences in Tsk compared to M1 exceeded 0.8°C across all ROIs, reaching 1.1°C in the anterior thigh regions (Figures 1 and 2). These findings underscore that 48 hours of rest may not suffice for complete athlete recovery. Thus, post-match activities should be carefully planned by coaching staff, taking into account observed fatigue and the prolonged recovery period needed.

Soccer clubs often implement standardized recovery protocols for all athletes following matches. However, individualized analysis of each athlete’s post-match physical condition is crucial for determining appropriate activities. Regarding CK analysis, the current value of 468.3 U/L (Figure 3) exceeds the average value of 388.2 U/L reported by Coelho [26]. Moreover, Tsk shows a similar pattern to CK analysis, whether in raw values or its individualized percentage (Figure 3).

## Other analyses

Several studies, including those by Côrte et al. [12] and Maior et al. [23], have noted symmetry between contralateral ROIs in both anterior and posterior views. This study similarly observed symmetry at all analysed moments, with the largest difference between ROIs being 0.2°C (Table 3). Notably, even after participating in official matches (M2), this symmetry persisted, potentially influencing limb balance. Although some athletes showed contralateral asymmetries across all moments without injury, these findings prompted the technical team to investigate further. This investigation aimed to discern whether these imbalances were indicative of underlying issues or part of an athlete’s normal individualized pattern.

Comparing ROIs between anterior and posterior views revealed significant differences, particularly notable at moments M1 and M3, with variations of approximately 0.4°C (Table 3). However, at M2, most ROIs showed no statistically significant difference between anterior and posterior views. This suggests that participation in soccer matches led to a balanced Tsk distribution between anterior and posterior regions.

The study’s limitations include incomplete data collection across all seasons and the absence of post-match data at extended time intervals. It would have been beneficial to include biochemical variables such as IL-6 and TNF-α. These findings are specific to professional soccer players, so caution is advised when applying them to other sports or non-professional athletes. The study spanned two seasons, potentially exposing the results to seasonal variability.

Although none of our participants reported any post-match injuries, there is a possibility of bias in Tsk measurements due to unreported contact injuries. Additionally, since Tsk measurements were conducted in the afternoon, circadian rhythm fluctuations could have affected the reliability of the result [29]. Future studies should consider extending the analysis up to 96 h after exercise to simulate soccer match conditions, and broaden the scope of biochemical variables assessed.

## CONCLUSIONS

This study on lower limb Tsk kinetics in soccer athletes at different time points across two seasons of the Brazilian Serie A championship concluded that Tsk significantly increased in all ROIs 24 h after matches, followed by a decrease 48 h later, though not returning to pre-match levels, indicating incomplete recovery during this period.

Tsk showed a temporal pattern similar to CK, with a significant moderate positive correlation observed when CK was analysed as a percentage. There is divergence and agreement in the findings from previous studies on CK and Tsk analysis. Therefore, we recommend that new research be conducted to test hypotheses that could either support or oppose our results. This will help clarify the role of thermography in monitoring elite soccer athletes.
